# Ionic Liquid-Based Immunization Patch for the Transdermal Delivery of Antigens

**DOI:** 10.3390/molecules29132995

**Published:** 2024-06-24

**Authors:** Rashedul Islam, Fahmida Habib Nabila, Rie Wakabayashi, Yoshirou Kawaguchi, Noriho Kamiya, Muhammad Moniruzzaman, Masahiro Goto

**Affiliations:** 1 Department of Applied Chemistry, Graduate School of Engineering, Kyushu University, 744 Motooka, Nishi-Ku, Fukuoka 819-0395, Japan; islam.rashedul.815@s.kyushu-u.ac.jp (R.I.); nabila.fahmida.871@s.kyushu-u.ac.jp (F.H.N.); kawaguchi.yoshirou.168@m.kyushu-u.ac.jp (Y.K.); kamiya.noriho.367@m.kyushu-u.ac.jp (N.K.); 2 Advanced Transdermal Drug Delivery System Center, Kyushu University, 744 Motooka, Nishi-Ku, Fukuoka 819-0395, Japan; 3 Division of Biotechnology, Center for Future Chemistry, Kyushu University, 744 Motooka, Nishi-Ku, Fukuoka 819-0395, Japan; 4 Department of Chemical Engineering, Universiti Teknologi PETRONAS, Seri Iskandar 32610, Perak, Malaysia; m.moniruzzaman@utp.edu.my

**Keywords:** transdermal drug delivery, ionic liquid, pressure-sensitive adhesive, IL-S/O patch, immunization

## Abstract

Herein, we report a transdermal patch prepared using an ionic liquid-based solid in oil (IL-S/O) nanodispersion and a pressure-sensitive adhesive (PSA) to deliver the macromolecular antigenic protein, ovalbumin (OVA). The IL-S/O nanodispersion and a PSA were first mixed at an equal weight ratio, then coated onto a release liner, and covered with a support film. To evaluate the effect of the PSA, three types of PSAs, DURO-TAK 87-4098, DURO-TAK 87-4287, and DURO-TAK 87-235A, were used to obtain the corresponding IL-S/O patches SP-4098, SP-4287, and SP-235A, respectively. The prepared IL-S/O patches were characterized for surface morphology, viscoelasticity, and moisture content. In vitro skin penetration and in vivo immunization studies of the IL-S/O patches were performed using Yucatan micropig skin and the C57BL/6NJc1 mice model, respectively. The SP-4098 and SP-4287 delivered 5.49-fold and 5.47-fold higher amounts of drug compared with the aqueous formulation. Although both patches delivered a similar amount of drug, SP-4287 was not detached fully from the release liner after 30 days, indicating low stability. Mice immunized with the OVA-containing SP-4098 produced a 10-fold increase in anti-OVA IgG compared with those treated with an aqueous formulation. These findings suggested that the IL-S/O patch may be a good platform for the transdermal delivery of antigen molecules.

## 1. Introduction

Immunization is one of the most successful medical programs worldwide and directly contributes to increasing life expectancy and saving millions of lives every year [1,2]. Therefore, the research and development of effective vaccines has received much attention. Most commercially available vaccines are usually administered to the muscle or subcutaneous layer by injection [3]. Injection as a mode of administration is associated with some disadvantages, including pain, needle injury, needle phobia, and the requirement for trained healthcare professionals [4]. Therefore, improving the route of administration is a major concern in ongoing vaccine research. Transdermal and oral delivery of vaccines are considered to be the best alternatives to injections because of the ease of administration and better bioavailability of these routes [5]. The presence of keratinocytes, Langerhans cells, dendritic cells, and mast cells makes the skin an immunocompetent and effective site for vaccination [4,6,7].

In addition to the immunocompetency of skin, transdermal immunization also offers several advantages over other routes of administration because transdermal immunization is painless and noninvasive and avoids first-pass metabolism [8,9,10]. Transdermal vaccination has been reported to produce equal and sometimes higher immunological responses than vaccination by injection [4]. However, there are also some challenges in the development of a transdermal drug delivery system (TDDS) that need to be overcome for successful drug delivery. The outermost layer of the skin, the stratum corneum (SC), imposes the main barrier to the delivery of therapeutic agents via the skin. The most superficial SC layer of the skin is composed of tightly packed lipids and corneocytes [11,12]. Because of the SC barrier, only small molecule drugs (MW < 500 g/mol), molecules with low lipophilicity, and therapeutics with water/octanol partition coefficients of 1–3 are appropriate for transdermal delivery [13,14]. Therefore, physical methods, such as microneedles, iontophoresis, and ultrasound, or chemical enhancers, for example, ethanol, esters, fatty acids, glycols, surfactants, phospholipids, and ionic liquids (ILs), are required to enhance the skin penetration of most molecules [15,16,17].

Recently, IL-mediated transdermal and topical drug delivery have emerged as excellent noninvasive delivery platforms for a wide range of biomolecules, including nucleic acids, proteins, and peptides [18,19,20,21]. Simply, ILs denote the molten organic salts at room temperature consisting of cations and anions. ILs typically have a melting point below 100 °C [22]. Transdermal formulations based on ILs are known to have increased drug delivery efficacy, better stability, and improved pharmacodynamics and pharmacokinetics compared with aqueous and conventional formulations [23,24]. ILs not only enhance skin penetration but can also solubilize water-insoluble drugs, such as acyclovir and paclitaxel [25,26]. Though some ILs are toxic, the recent development of biocompatible ILs allows them to be used in pharmaceutical applications [27,28]. Widely studied IL formulations for TDDSs are IL in oil (IL/O) microemulsions [29], solid in oil (S/O) dispersions [30], ethosomes [31], and oil in water (O/W) emulsions [32]. Most of these formulations are dispersed in an oil phase or organic solvent and show low viscosity. This low viscosity means that these formulations are not well retained on the skin and require additional support for skin application. To address this issue, we have recently reported a new transdermal patch system that incorporated an IL/O formulation in a pressure-sensitive adhesive (PSA) with adequate viscosity, which could be applied directly to the skin without requiring any additional support [33]. This IL-based transdermal patch provided enhanced delivery of the antiviral drug acyclovir compared to conventional formulation. This enhanced delivery indicated that the incorporation of an IL-based formulation in a PSA may pave the way for an effective new TDDS. These results encouraged us to explore the incorporation of IL-based S/O formulations in different types of PSA. Our aim was to deliver the antigenic protein ovalbumin (OVA), which is a hydrophilic drug with a high molecular weight, that is more than 189 times higher than acyclovir (acyclovir, MW: 225 g/mol; OVA, MW: ca. 42.7 kDa).

In the present study, we developed an IL-based transdermal patch for the delivery of a macromolecule (OVA) and investigated the immunization efficacy in a murine model. OVA is well known to induce the production of immunoglobulin G (IgG), including subclasses IgG1 and IgG2a [34]. We selected a biocompatible IL containing 1,2-dimyristoyl-sn-glycero-3 ethyl-phosphocholine (EDMPC) and linoleic acid (Lin) as the cation and anion components, respectively. An IL-S/O nanodispersion containing OVA was incorporated in different PSAs to prepare IL-S/O patches. The surface morphology and adhesion properties of the patches were investigated using a scanning electron microscope and rheometer, respectively. In addition, the stability of the patches was studied in terms of both the physical and chemical properties to assess the consistency of the patches over time. In vitro skin permeation and in vivo immunization using the patches were also investigated to evaluate the antibody production efficiency. The in vivo immunization results indicated that the IL-S/O patch could produce an adequate immune response.

## 2. Results and Discussion

### 2.1. Preparation of the IL-S/O Patches

**Figure 1:**
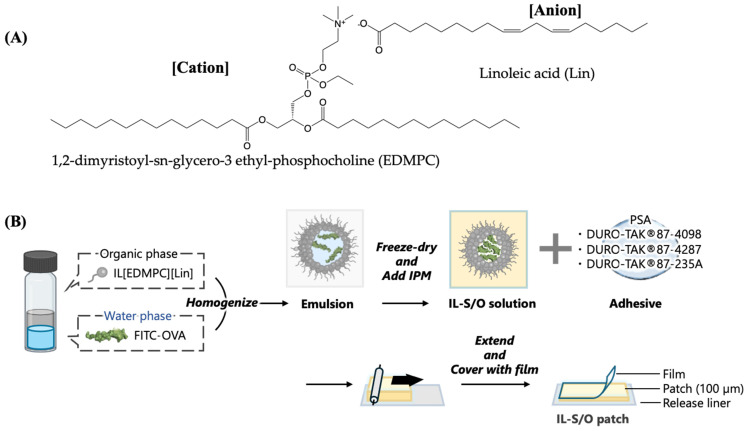
(**A**) Structure of Ionic Liquid (IL) and (**B**) general outline of the IL-S/O patch preparation.

The IL-S/O nanodispersion was prepared using 10% (w/v) of the biocompatible IL [EDMPC][Lin] dispersed in isopropyl myristate (IPM). Three different IL-S/O patches were prepared by combining the [EDMPC][Lin]-based IL-S/O nanodispersion with three PSAs with different chemical compositions. The preparation procedure of the patch is illustrated in Figure 1B. 

The details of the PSAs are summarized in Table 1. We selected [EDMPC][Lin] because it has proven biocompatibility and excellent drug delivery capacity through the skin [19,20,35]. Our previous study of an IL/O patch indicated that a thin patch (100 μm) had a better penetration rate than thicker patches (200–500 μm) [33]. Therefore, in the present study, we prepared only 100 μm patches using the three different combinations.

### 2.2. Characterization of the IL-S/O Patches

Two of the three prepared patches, SP-4098 and SP-4287, were able to be separated from the release liner, and all of the material remained attached to the support film. 

**Figure 2:**
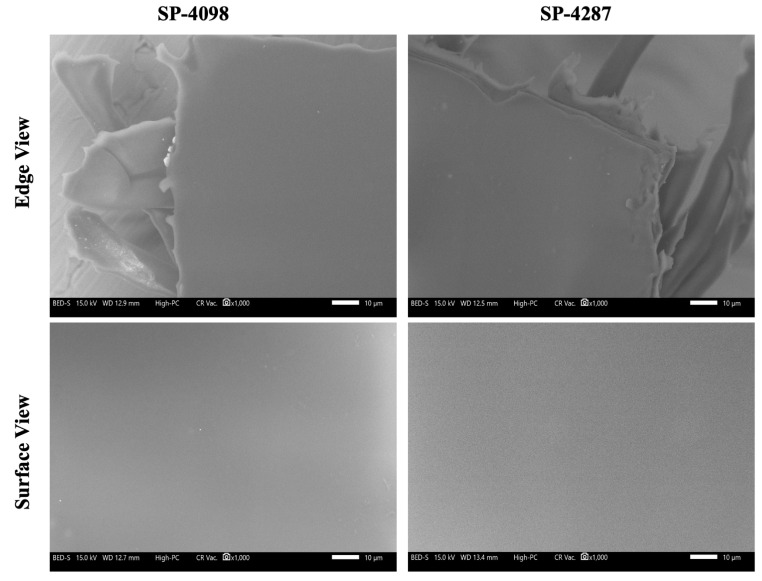
Scanning electronic microscopy (SEM) images of SP-4098 and SP-4287. The magnification is 1000× and the applied voltage was 15.0 kV. The scale bar is 10 µm.

However, SP-235A did not fully detach from the liner, a phenomenon termed patch failure [36]. To understand the details of this failure, different ratios of IL-S/O and PSA need to be investigated with various evaporation temperatures. Only the two suitable patches were further characterized. Patches were prepared with fluorescein isothiocyanate (FITC) conjugated OVA or unlabeled OVA. Upon visual inspection, the non-FITC OVA-containing patches were found to be transparent while the FITC-containing patches were a yellowish color (Figure S1). This result indicated that neither the incorporation of the IL-S/O in PSA nor changing the type of PSA impacted the visual appearances of the patches. An ideal transdermal patch should have a smooth surface [37]. However, some transdermal patches may have rough surfaces depending on the type of PSA [38]. The surface morphology of SP-4098 and SP-4287 were investigated by scanning electron microscopy (SEM) and found to be smooth (Figure 2). No notable differences were observed in the SEM micrographs of SP-4098 and SP-4287. Moreover, no visible pores or separations were observed in the matrix of either patch. These findings were similar to the results for previous IL and non-IL-based transdermal patches, in which no pore formation, phase separation, or roughness were observed [33,39].

**Figure 3:**
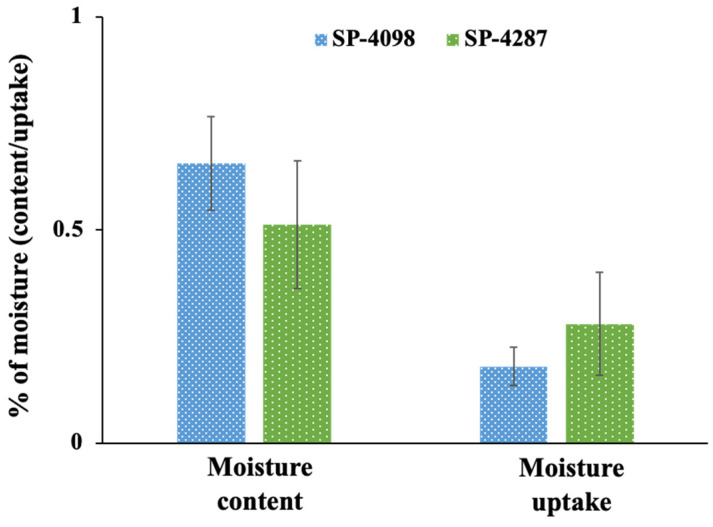
Percentage of moisture content and moisture uptake by SP-4098 and SP-4287.

In addition, both patches were assessed for moisture uptake and moisture content parameters. The moisture content for both SP-4098 and SP-4287 was found to be <1.0% and the moisture absorption was <0.5%. (Figure 3). The differences in the moisture content and uptake between the two patches were negligible. PSAs with a functional group interact with drug molecules and surfactants present in a patch and play a crucial role in the thermophysical properties of patches [40]. Low values for both moisture absorption and moisture content are desirable for an ideal transdermal patch [38,41].

### 2.3. Rheological Properties of the IL-S/O Patches

**Figure 4:**
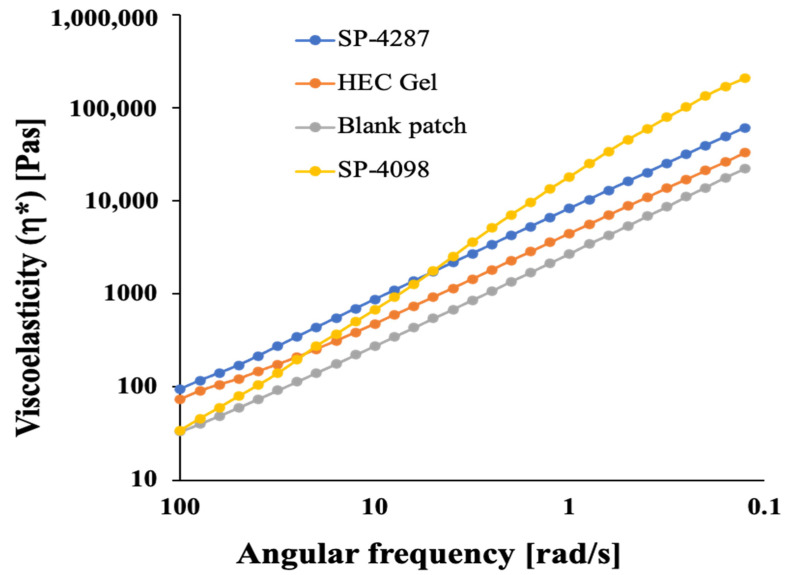
Viscoelastic properties of the IL-S/O patches; 8.5% HEC gel was used as a control gel.

The adhesion properties of the patches were evaluated using a rheometer. Mixing the IL-S/O nanodispersion with an adhesive improved the viscoelasticity of the transdermal patch. The viscoelasticity (η*) of SP-4098 and SP-4287 was compared with that of a blank patch, and hydroxyethyl cellulose (HEC) gel was used as a control. HEC is a commonly used gelling agent for transdermal patches or gel; therefore, it was used as a control to compare the rheological properties of IL-S/O patches [42,43]. Both SP-4098 and SP-4287 showed higher viscoelasticity than that of the control and blank patches at all frequencies. Although SP-4098 showed a higher elasticity than SP-4287 at a lower frequency, at a higher frequency, SP-4287 showed more adhesion properties than SP-4098 (Figure 4). The blank patch was found to be less viscous than the HEC gel, indicating that the incorporation of an IL-S/O in PSA improved the adhesion of the patches. The blank patch contains only PSA and IPM while IL-S/O patches contain IL, PSA, and IPM. Usually, ILs are more viscous than conventional solvents, and this viscous property leads to the increased adhesion of IL-S/O patches [44]. Under shear strain, the η* value of the IL-S/O patches started decreasing at a higher frequency, indicating that disruption of the molecular orientation of the patches had occurred. The storage modulus (G′) and loss modulus (G″) values are directly related to the adhesion performance of a substance onto the surface of a substrate (e.g., skin). A good adhesive has a low G′ value at a low angular frequency (bonding) and a G′ value that is ≥the G″ value at a higher frequency (debonding) [45]. The observed values of G′ for both SP-4098 and SP-4287 were found to be higher than G″ at all angular frequencies (Figure S2).

These findings indicated that both patches had good adhesion performance and closely resembled other PSA-based transdermal patches [46,47].

### 2.4. Stability of the IL-S/O Patches

**Figure 5:**
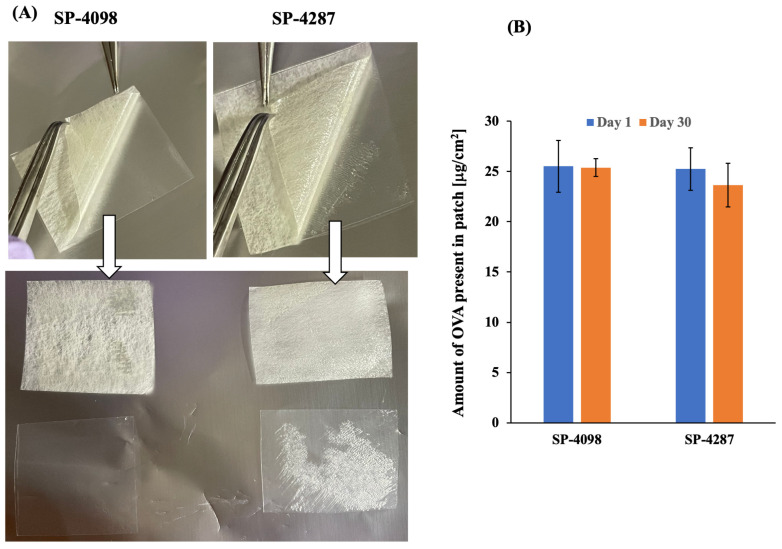
Stability of the IL-S/O patches; (**A**) illustrates the peeling characteristics of the IL-S/O patches after 30 days and (**B**) shows the amount of OVA present in the patches initially and after 30 days.

It is important for a pharmaceutical formulation to have good stability. The efficacy of a drug greatly relies on the stability of the formulation. The stability of [EDMPC][Lin] IL-based formulations has been widely studied under different conditions [19,30]. Therefore, we carried out a stability study of the IL-S/O patches only in terms of physical appearance, surface morphology, and drug content for 30 days at room temperature. After 30 days, no changes were observed by visual inspection of SP-4098 and SP-4287 (Figure S3). However, the peelable nature of SP-4287 was decreased after 30 days and a portion of the material was retained on the release liner (Figure 5A). These findings were supported by the surface morphology observations by SEM on the 30th day (Figure S4). The fully separated part of SP-4287 was examined by SEM and some non-uniformity was observed, indicating deterioration of the patch, which was not observed for SP-4098.

The drug, OVA, in the IL-S/O patches, was quantified after 1 month and compared with the initial amount of the OVA present in the two patches. Initially, SP-4098 and SP-4287 contained 25.50 ± 2.56 and 25.23 ± 2.13 μg/cm^2^ of the drug, respectively. After 30 days, although the drug content was not changed significantly, SP-4287 contained 23.65 ± 0.89 μg/cm^2^ while SP-4098 contained 25.38 ± 2.17 μg/cm^2^ of the drug. These findings suggested that SP-4098 was more suitable for transdermal application than SP-4298.

### 2.5. In Vitro Skin Permeation Study

**Figure 6:**
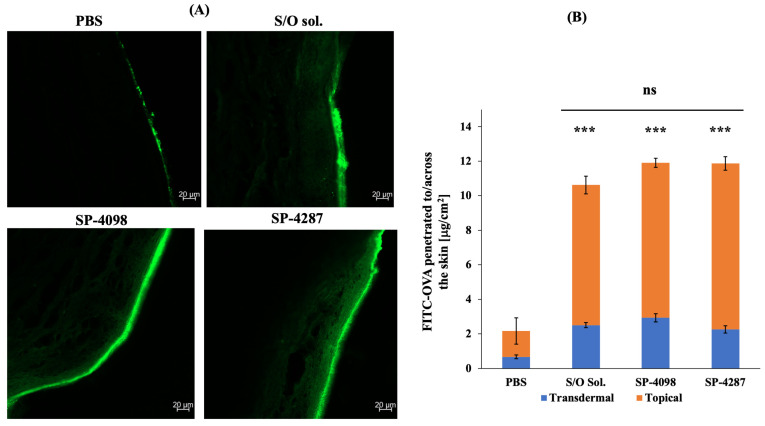
Penetration of FITC-OVA in YMP skin after 24 h of administration (**A**) and amounts of OVA delivered transdermally and topically after 24 h (**B**). All data presented are the means of three samples ± SD. *** *p* < 0.001.

The skin penetration ability of 100 μm thick IL-S/O patches was evaluated using Yucatan micropig (YMP) skin. As well as SP-4098 and SP-4287, IL-S/O and phosphate buffer saline (PBS) solutions containing equal amounts of the drug were evaluated using Franz diffusion cells (FDCs) at 32.5 °C for 24 h. Because a 100 μm thick patch was found to be better than thicker patches in our previous study [33], both SP-4098 and SP-4287 were prepared only as 100 μm thick patches. We investigated the distribution of FITC-OVA within the skin after delivery via different formulations. Most of the FITC-OVA was accumulated in the SC layer and no fluorescent signal was detected in the dermal and epidermal layers of skin treated with an aqueous solution of FITC-OVA. In contrast, fluorescent signals were observed throughout the skin layers after treatment with IL-S/O nanodispersion, SP-4098, and SP-4287 (Figure 6A). After administration for 24 h, the drug content was quantified (Figure 6B). 

The amount of drug in the receiver phase after 24 h was 2.93 ± 0.24, 2.26 ± 0.21, 2.5 ± 0.15, and 0.67 ± 0.11 μg/cm^2^ for SP-4098, SP-4287, IL-S/O nanodispersion, and PBS solution, respectively. A higher amount of drug was delivered into the skin, 8.98 ± 0.27, 9.61 ± 0.39, 8.12 ± 0.51, and 1.5 ± 0.76 for SP-4098, SP-4287, IL-S/O nanodispersion, and PBS solution, respectively. In terms of total drug delivery, SP-4098, SP-4287, and IL-S/O nanodispersion delivered similar amounts. However, compared with the PBS solution, SP-4098, SP-4287, and IL-S/O nanodispersion delivered 5.49-, 5.47-, and 4.89-fold higher amounts of the drug. The presence of the lipid-based IL in the IL-S/O patches caused the increased drug delivery because of its hydrophobicity and ability to interact with the skin. Aqueous solutions cannot facilitate the penetration of large drugs, such as OVA; however, formulations containing hydrophobic ILs can enhance the skin penetration of such drugs. The skin penetration ability of the IL [EDMPC][Lin] has previously been reported by Uddin et al. [20]. The present study demonstrated that the skin penetration of the [EDMPC][Lin]-based IL-S/O patches with improved viscoelasticity was not compromised. The viscoelasticity of a formulation is directly connected to the drug diffusion and delivery. Less viscous formulations are not well retained on the skin, resulting in reduced drug delivery efficacy. However, highly viscous formulations often result in less drug diffusion from the depot compared with less viscous formulations. Therefore, formulations with moderate viscosity, which does not hinder skin penetration, are desirable [48].

### 2.6. Effect of IL-S/O Patches on the SC Layer

SC imposes the primary barrier to the penetration of drugs and other foreign particles through the skin. The distinctive alignment of keratin proteins and hydrophobic lipids (fatty acids, cholesterol, and ceramides) is responsible for the barrier function of the SC layer [49,50]. The molecular mechanism underlying the facilitation of the drug delivery through the compact SC layer was studied by Fourier-transform infrared (FTIR) spectroscopy. 

**Figure 7:**
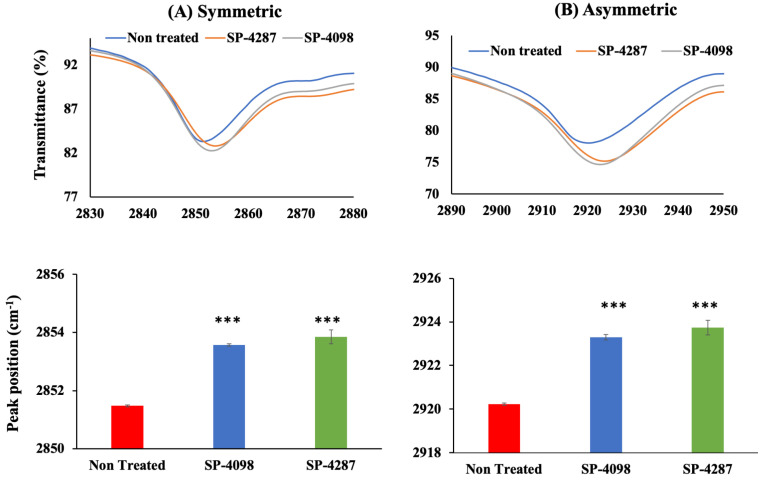
FTIR spectroscopic investigation of the SC layer after treatment with the IL-S/O patches. Spectra and peak position changes for (**A**) symmetric CH_2_ stretches and (**B**) asymmetric CH_2_ stretches. All data are the means of three samples ± SD. *** *p* < 0.001.

Vibrational changes in the lipid layer, specifically symmetric and asymmetric CH_2_ stretching at approximately 2850 and 2920 cm^−1^, respectively, are attributed to the structural disorganization of the SC layer [51,52]. The SC layer separated from YMP skin was treated with SP-4098 and SP-4287, FTIR measurements were conducted, and the results were compared with those for a non-treated SC sheet as a control. The FTIR spectra of the control SC layer showed symmetric and asymmetric CH_2_ bond stretching at approximately 2851 and 2920 cm^−1^, respectively. SP-4098- and SP-4287-treated samples generated a red shift and showed characteristic peaks at approximately 2853 and 2923 cm^−1^ for symmetric and asymmetric CH_2_ stretches, respectively (Figure 7). The changes observed in the treated samples were produced by the presence of the ILs in the IL-S/O patches. As we have previously reported, Lin, the anion in the IL used in the patches can alter the molecular orientation of the SC layer and facilitate the penetration of drugs through the skin [19]. The above results indicated that the IL-S/O patch was capable of dissociating the lipid nanostructure to assist in the delivery of OVA through the skin.

### 2.7. In Vitro Skin Irritation Study

The skin irritation profile of the IL-S/O patches was investigated using the LabCyte EPI-MODEL 24. The biocompatibility of the [EDMPC][Lin] and [EDMPC][Lin] formulations containing OVA, leuprolide, and oligonucleotides has been extensively studied [19,30]. PSAs have also been used in transdermal formulations with no reported safety issues [47,53,54].

**Figure 8:**
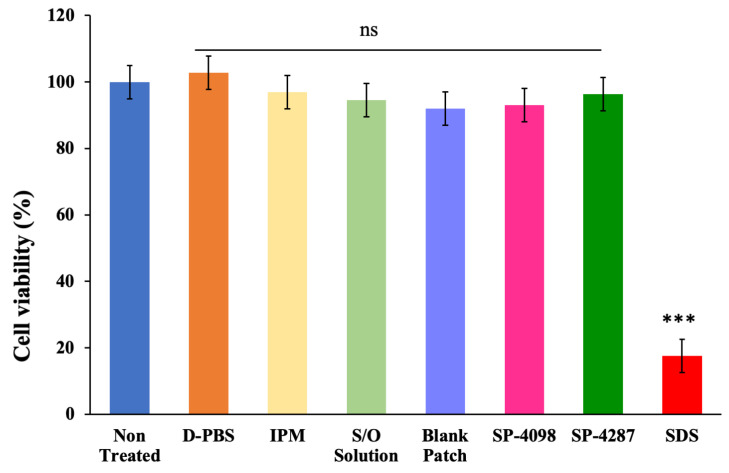
Results of the in vitro skin irritation study of the IL-S/O patches using the EPI-MODEL 24. Data are presented as the means of three samples ± SD. ns: non-significant, *** *p* < 0.001.

However, the biocompatibility of a combination of PSA and an OVA-containing IL-S/O formulation has not previously been investigated. Therefore, the skin irritation profiles of an IL-S/O nanodispersion, blank patch, SP-4098, and SP-4287 were evaluated along with IPM and Dulbecco’s Phosphate-Buffered Saline (D-PBS) as negative controls and 5.0% sodium dodecyl sulfate (SDS) as a positive control. All the test samples were also compared with a non-treated healthy control. The cell viability of the negative controls, the known non-irritants IPM and D-PBS, were 102.76 ± 2.55 % and 96.92 ± 8.68 %, which indicated that these compounds did not negatively affect the cell viability. SDS significantly reduced the cell viability to below 20%. The cell viabilities of the blank patch, SP-4098, and SP-4287 were >90% and the differences with the negative control were negligible (Figure 8). According to the Organisation for Economic Co-operation and Development (OECD) guidelines for in vitro skin irritation studies on reconstructed human epidermal tissue, any test sample that decreases the cell viability to below 50% is considered an irritant to the skin. If the cell viability is more than 50%, a sample is regarded as safe. In our experiment, the cell viability of the test samples was higher than the OECD limit, which indicated a good safety profile for the IL-S/O patches. This finding was consistent with previous reports on the biocompatibility of transdermal formulations using 3D cell lines [20,33].

### 2.8. In Vivo Immunization Study

**Figure 9:**
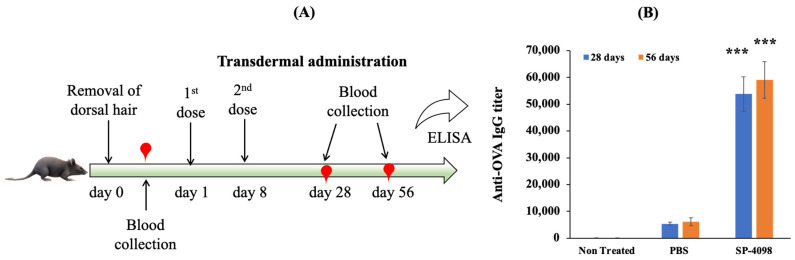
In vivo immunization study design (**A**) and anti-OVA IgG titer after 28 and 56 days of immunization (**B**). Data are presented as the means of five samples ± SD. *** *p* < 0.001.

Finally, we investigated the immune response in a murine model. Mice were divided into three groups and treated with SP-4098 and PBS (transdermal), and one group was left as a non-treated group. Mice were immunized twice with OVA at a 7-day interval. We selected SP-4098 for the in vivo immunization study as SP-4098 was more stable than SP-4287. Sera were collected before immunization and 28 days and 56 days after immunization. The OVA-specific IgG titer was quantified by ELISA. The antibody titer is defined as the reverse dilution at which the optical density obtained by ELISA of immunized mice is equal to that of diluted samples with pre-immunization sera [55]. Compared with transdermal PBS, SP-4098 produced a significantly (*p* < 0.001) higher amount of anti-OVA IgG 28 days after the first administration (Figure 9). The IgG titer tended to increase in the SP-4098 treated group after 56 days of immunization indicating the production of a stable immune response. The IL present in the IL-S/O patch enhanced the penetration of the OVA antigen through the skin, enabling the presentation of OVA to antigen-presenting cells (APCs) in the skin, which promoted the production of anti-OVA IgG. These results also indicated that the encapsulated drug, OVA, retained antigenic properties after incorporation into the patches and therefore could induce an immune response in the murine model.

SP-4098 could successfully deliver the antigenic protein OVA, resulting in the production of a significant level of anti-OVA-specific IgG without any pretreatment of the skin. The expression of IgG is also connected with cancer metastasis in various cancers (colon, lung, and pancreatic cancer) [56,57,58]. OVA is a model anticancer agent that is used to suppress tumor growth [59]. The transdermal patch developed in the present study could be a good option for the delivery of antigenic molecules to treat cancer. Further studies are needed to understand the detailed mechanisms of the developed transdermal patch for use in cancer immunotherapy.

## 3. Materials and Methods

### 3.1. Materials

IPM, 1,2-dimyristoyl-sn-glyecero-3-phosphocholine (DMPC), ethyl trifluoromethane sulfonate, span-20, Tween-20, and Lin were procured from Tokyo Chemical Industry Ltd. (Tokyo, Japan). Isopropyl alcohol (IPA), acetonitrile, methanol, ethanol, chloroform, and 3,3′,5,5′-tetramethylbenzidine (TMB) solution for microwell were purchased from Fujifilm Wako Pure Chemical Corporation (Osaka, Japan). 3-(4,5-Dimethyl-2-thiazolyl)-2,5-diphenyl-2*H*-tetrazolium bromide (MTT) and FITC were purchased from Dojindo Laboraories Inc. (Kumamoto, Japan). OVA from chicken egg white and bovine serum albumin (BSA) were purchased from Sigma-Aldrich (St. Louis, MO, USA). DURO-TAK (87-4098, 87-4287, 87-235A) was a gift from Henkel Corporation, Minoh, Japan.

### 3.2. Methodology

#### 3.2.1. Synthesis of IL

The IL [EDMPC][Lin] was synthesized and characterized according to a previously published report [35]. In brief, equimolar amounts of 1,2-dimyristoyl-sn-glycero-3-phosphatidylcholine and ethyl trifluoromethanesulfonate were mixed and reacted at 45 °C overnight under a continuous flow of nitrogen gas. The produced EDMPC was reacted with HCl to obtain EDMPC-Cl. Finally, EDMPC-Cl and Lin were mixed in a molar ratio of 1:1 in chloroform and allowed to react for 12 h at 45 °C with a constant supply of nitrogen gas. The chloroform was then evaporated using a rotary evaporator. The IL was then characterized by FTIR and proton NMR.

#### 3.2.2. Preparation of IL-S/O Nanodispersion

The IL-mediated IL-S/O nanodispersion was prepared according to previously published reports with slight modifications [30]. In brief, 100 mg of IL was dissolved in 4 mL of cyclohexane, and 5 mg of FITC-OVA was dissolved in 1 mL of Milli-Q water. The solutions were combined and homogenized at 26,000 rpm for 2 min using a mechanical homogenizer (PT-MR 3100 D; Polytron, KINEMATICA, Tokyo, Japan). The cyclohexane and water from the emulsion were removed by freeze-drying (FDS 2000, EYELA, Tokyo, Japan) for 24 h. The resulting IL drug mixture was then dispersed in 1 mL of IPM. The particle size distribution of this IL-S/O was measured by dynamic light scattering (Zetasizer Nano ZS, Malvern Instruments, Malvern, UK).

#### 3.2.3. Preparation of Transdermal IL-S/O Patches

The IL-S/O patches were prepared using the solvent evaporation method as described in our previous study [33]. Equal amounts of the IL-S/O nanodispersion and different PSAs were mixed at a 1:1 ratio to prepare the IL-S/O patches. The IL-S/O nanodispersion and a PSA were measured and blended in a glass vial by rigorous vortexing to prepare a uniform admixture. The names and compositions of the PSAs are listed in Table S1. The homogenous IL-S/O-PSA mixture was then dispensed onto a polyethylene terephthalate release liner and coated using a K control coater (RK Prints, Royston, UK). The organic solvent present in the IL-S/O patches was then removed by evaporation at 60 °C for 30 min in a vacuum oven (Ishii Laboratory Works Co. Ltd., Osaka, Japan) with a cold trap unit (Uni Trap UT-1000, EYELA, Bohemia, NY, USA). Once evaporated, the IL-S/O patches were covered by a support film made of polyethylene terephthalate (PET) and stored at room temperature until use.

#### 3.2.4. Characterization of the IL-S/O Patches

The IL-S/O patches were first examined for compatibility between each of the components i.e., the IL-S/O nanodispersion, five different PSAs, release liner, and support film. Successfully prepared IL-S/O patches were then examined for visual appearance, surface morphology, viscoelasticity, moisture content, moisture uptake, and drug content. A benchtop scanning electronic microscope (JCM-7000, NeoScope™, Tokyo, Japan) was used to evaluate the surface morphology of the IL-S/O patches. The experiment was carried out in low vacuum mode at 15 kV. Moisture content and uptake were measured following the protocols described by Gadag et al. [60]. The IL-S/O patches were cut into pieces (~1.0 cm^2^), the weights were measured, and the pieces were placed in a desiccator containing fused calcium chloride for up to 24 h. After 24 h, the patches were collected from the desiccator and re-weighed. The moisture content was calculated according to the following formula:

$$Moisture\;content\;(\%)=\frac{Initial\;weight-Final\;weight}{Final\;weight}\times 100$$

For moisture uptake, the IL-S/O patch pieces were weighed and placed in a desiccator with saturated sodium chloride solution, at a relative humidity of 75% ± 5%. The IL-S/O patches were weighed at 24, 48, and 72 h. Finally, the moisture content (%) was calculated using the formula given below.

$$Moisture\;uptake\;(\%)=\frac{Final\;weight-Initial\;weight}{Initial\;weight}\times 100$$

The viscoelastic properties of the IL-S/O patches were measured using a rheometer (MCR501, Anton Paar, Graz, Austria) fitted with a 25 mm cone plate. The prepared IL-S/O patches were mounted on a disposable dish. The data were curated by gradually raising the frequency from 0.1 to 100 Hz. Additionally, a control gel consisting of 8.5% hydroxyethyl cellulose was also prepared to compare the gelling properties of the IL-S/O patches [43].

#### 3.2.5. Determination of Entrapped Drug in IL-S/O patches

To measure the amount of drug present in the patches, 1 cm^2^ samples of the patches were cut from three different areas and dissolved in a mixture of water and ethanol (3:7 *v*/*v*). Ultrasonication and vortexing methods were used to fully extract the drug from the patch pieces. The content of FITC-OVA in each piece was then measured using a multiple microplate reader at 485–535 nm with appropriate dilution.

#### 3.2.6. Stability Study of the IL-S/O patches

A stability study of the prepared IL-S/O patches was carried out in terms of physical appearance, surface morphology, and drug content. The patches were stored at room temperature for 30 days. Physical appearance was observed and photographed after 30 days. The surface morphology of the IL-S/O patch was investigated by SEM. Drug content was quantified by following the methods mentioned in Section 3.2.5. 

#### 3.2.7. In Vitro Skin Permeation Study

The skin penetration of the IL-S/O patches was investigated using FDC with YMP (The Jackson Laboratory, Bar Harbor, ME, USA) skin following a previously reported protocol [61]. The extra fat layer of the skin was removed using a surgical stainless-steel blade and the skin was chopped into 1.5 cm *×* 1.5 cm pieces. The receiver phase of the FDCs was filled with 5 mL of PBS and the skin was clamped on the FDCs with the SC layer facing upwards. The IL-S/O patches (1 cm^2^) were applied to the skin in the donor phase. The analysis was conducted at 32.5 °C. After 24 h, the samples from the receiver phase were collected and the amount of transdermally delivered FITC-OVA was quantified using a multiple microplate reader at 485–535 nm. To quantify the topical delivery, after 24 h, the skin was washed three times with 20% ethanol and cut into pieces to extract the FITC-OVA from the skin. The extraction was carried out in a mixture (*v*/*v*) of 50% PBS, 25% methanol, and 25% acetonitrile overnight with continuous shaking, then the fluorescence intensity was measured.

To investigate the deepness of the skin penetration, YMP skin was washed with 20% ethanol and blocked in the embedding medium for frozen tissue specimens to ensure optimal cutting temperature (OCT) (Sakura Fine Tech, Tokyo, Japan) at –80 °C. This immobilized skin tissue was then sliced into 20 μm cross-sections with the help of a cryostat microtome (Leica CM186OUV, Leica Biosystems, Wetzlar, Germany) and transferred to a glass slide. Images of the skin sections were obtained using a confocal laser scanning microscope (LSM900, Carl Zeiss, Jena, Germany).

#### 3.2.8. Effect of IL-S/O Patches on the SC Layer

The SC layer was prepared according to previously reported methods with slight modifications [62,63]. Frozen pig skin was thawed and the fat layer was removed. To separate the epidermal layer, the skin was heated at 60 °C for 10 min. The collected epidermal layer was then treated with 0.25% trypsin and 1 mM EDTA for 24 h at room temperature. Then, the extra tissue debris from the SC layer was removed by washing with water and the SC layer was dried for 24 h at ambient temperature. The SC layer was cut into the desired size (1 cm × 1 cm) and IL-S/O patches were applied for 24 h at 32.5 °C. This experiment was carried out in an incubator (EYELA SLI-220, Shanghai, China) to maintain a constant temperature. The patches were then removed from the SC layer, washed with 20% ethanol, and the SC layer was allowed to dry for 24 h. Finally, the FTIR spectra were measured using an ATR-FTIR (Perkin Elmer, Shelton, CT, USA) at 1 cm^−1^ resolution for 16 scans. A non-treated SC layer was used as the control.

#### 3.2.9. In Vitro Skin Irritation Study

LabCyte EPI-MODEL, a 3D artificial model of human epidermal tissue was used to conduct the in vitro skin irritation study of the IL-S/O patch. As described in the manufacturer’s protocols, 25 μL or 25 mg of test samples was added onto the surface of the 3D epidermal cell line. The treated epidermal tissues were incubated at 37 °C and 5% CO_2_ for 15 min. Then, the tissue was washed with D-PBS to remove the test sample and incubated for 3 h in an MTT assay medium (0.5 mg/mL MTT) for formazan production. IPA was used as a solvent to extract the formazan produced during the reaction. The optical density was obtained at 570 and 650 nm. Using IPA as the negative control, the number of live cells was calculated using the following formula.

$$\mathrm{Cell}\mathrm{viability}\;(\%)=\frac{\left(Absorbanc\;of\;test\;sample-Absorbance\;of\;blank\;\right)}{\left(Absorbance\;of\;negative\;control-Absorbance\;of\;blank\right)}\times 100$$

#### 3.2.10. In Vivo Immunization and Antibody Measurement

For the in vivo immunization, C57BL/6NJcl female mice (6–7 weeks old, 19 ± 3 g) were used. Mice were kept in an animal room for 7 days after purchase to adjust to the new environment. A test solution containing 100 μg of OVA (in 100 μL of PBS) was administered by homemade transdermal patch onto the dorsal skin of mice. A 2 cm × 2 cm IL-S/O patch containing 100 μg of OVA was also applied to the dorsal skin of the mice. The transdermal PBS and IL-S/O patches were removed after application for 24 h. Each group contained five mice and was treated with a formulation on days 1 and 8. Blood was collected on the 28th and 56th days and the anti-OVA IgG titer was quantified by ELISA. Details of the ELISA are provided in the Supplementary Information.

#### 3.2.11. Statistical Analysis

Statistical analysis of all data was carried out using GraphPad Prism software version 6.05. A *p*-value < 0.05 was considered as statistically significant. All data are presented here as the mean ± standard deviation.

## 4. Conclusions

We have developed an IL-based transdermal patch system incorporating an IL-S/O nanodispersion and PSA, which could effectively deliver a biomacromolecule through the skin. The patches showed favorable adhesive properties for transdermal administration. A skin permeation study demonstrated that the patches could deliver OVA more effectively than an aqueous formulation. SP-4098 exhibited superior stability compared with SP-4287. In vivo immunization with SP-4287 generated a substantial amount of IgG antibodies, which indicated the proper presentation of the OVA antigen to APCs. This study provides a new strategy for the transdermal delivery of high molecular weight drugs and other biologically active molecules. This study is a primary investigation that indicates the capability of producing immune responses in vivo. Further in-depth studies could be carried out to explore immunization efficacy and the mechanism of action of IL-S/O patches in detail.

## Figures and Tables

**Table 1:** Characteristics of the PSAs and corresponding IL-S/O patches.

Properties of PSAs						Patch Properties	
Name of PSA	Polymer Name	Vinyl Acetate	Functional Group	Crosslinker	IL-S/O: PSA	Patch Name	Comment
DURO-TAK 87-4098	Acrylate copolymers	Yes	None	N/A	1:1	SP-4098	* Suitable
DURO-TAK 87-4287	Acrylate copolymers	Yes	-OH	No	1:1	SP-4287	* Suitable
DURO-TAK 87-235A	Acrylate copolymers	No	-COOH	No	1:1	SP-235A	** Not suitable

* Patch can be fully separated from the release liner, ** Patch is not fully separated from the release liner.

## Data Availability

All data curated or analyzed during this study are presented in the article.

## Supplementary Materials

The following supporting information can be downloaded at: https://www.mdpi.com/article/10.3390/molecules29132995/s1, Figure S1: Photographs of IL-S/O patches. (A) With FITC-OVA and (B) with OVA; Figure S2: Storage modulus and loss modulus of SP-4098 and SP-4287; Figure S3: Physical appearance of IL-S/O patches on days 1 and 30; Figure S4: Surface morphology of IL-S/O patches by SEM after 30 days. Figure S5: ^1^H-NMR of ionic liquid (IL). Spectra was obtained by JEOL ECZ400S 400MHZ NMR, Tokyo, Japan. Figure S6: FTIR spectra of 1,2-dimyristoyl-sn-glycero-3 ethyl-phosphocholine (EDMPC), linoleic acid (Lin), and [EDMPC][Lin] IL. Figure S7: Particle size distribution of IL-S/O. The measured particle size is 174 ± 4.945 nm with a polydispersity index of 0.295 ± 0.049.
