# Supplementary material for: Ionic Liquid-Based Immunization Patch for the Transdermal Delivery of Antigens
Source: Molecules. 2024 Jun 24;29(13):2995. doi: 10.3390/molecules29132995 (PMC11243093; doi:10.3390/molecules29132995)
Supplement: Supplementary file 1 [file molecules-29-02995-s001.zip › molecules-3028331-supplementary.pdf]

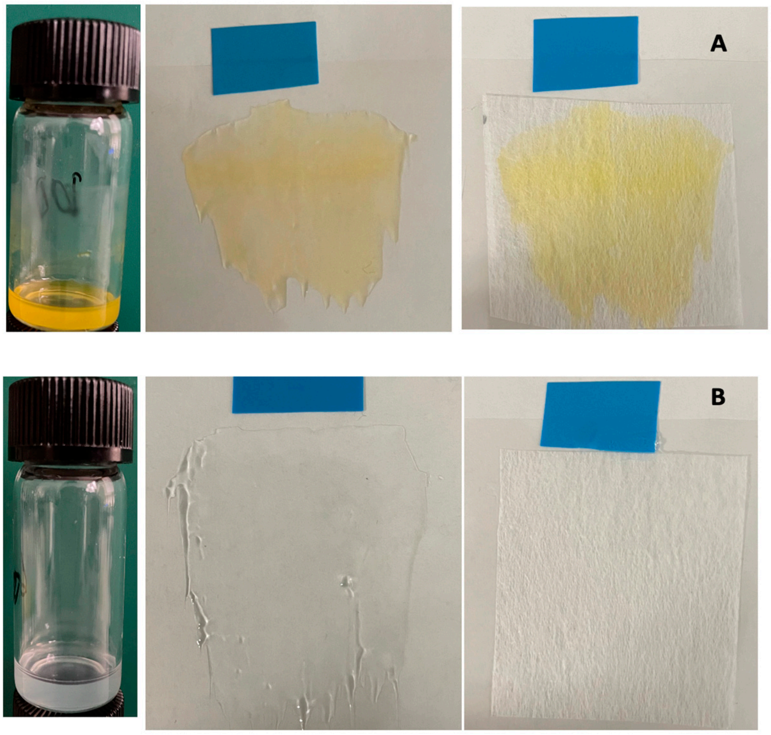

Figure S1. Photographs of IL-S/O patches. (A) With FITC-OVA and (B) with OVA.

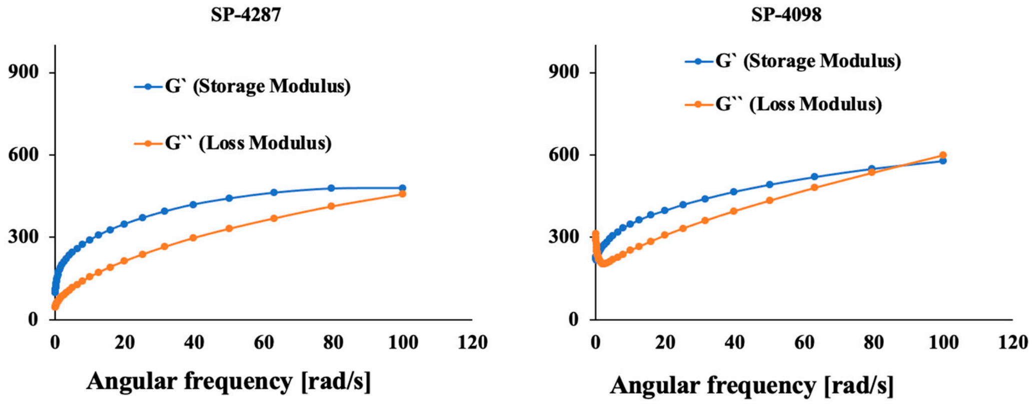

Figure S2. Storage modulus and loss modulus of SP-4098 and SP-4287.

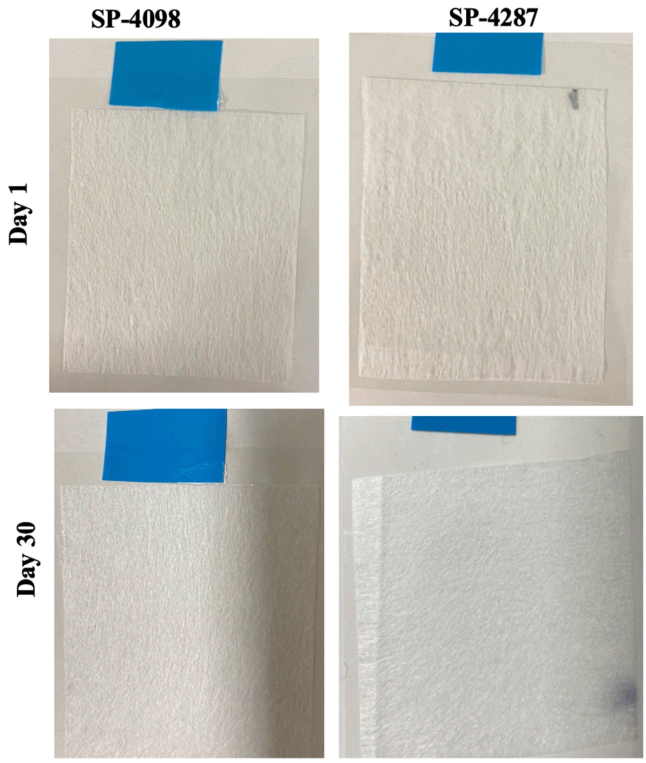

Figure S3. Physical appearance of IL-S/O patches on days 1 and 30.

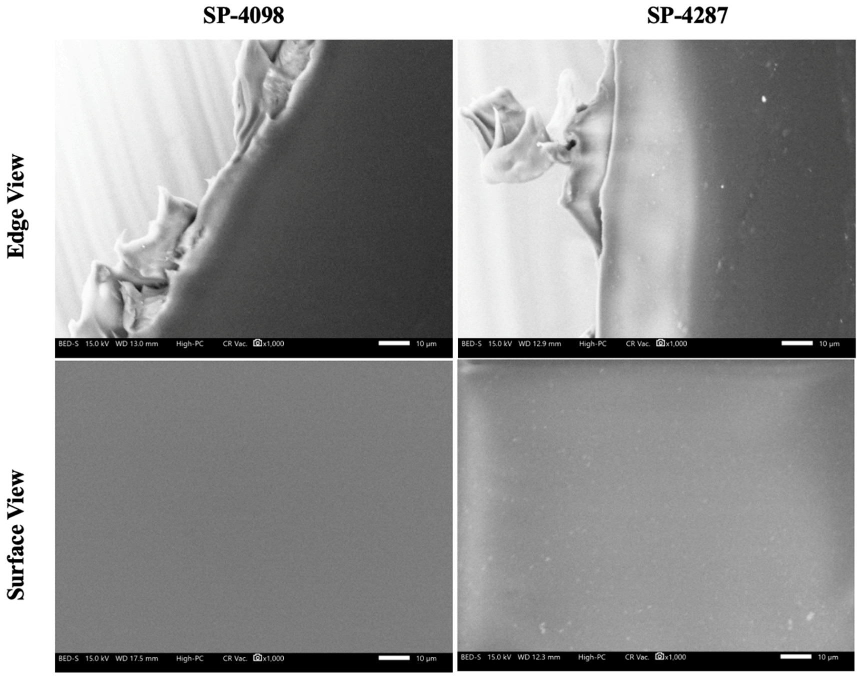

Figure S4. Surface morphology study of IL-S/O patches by SEM after 30 days.

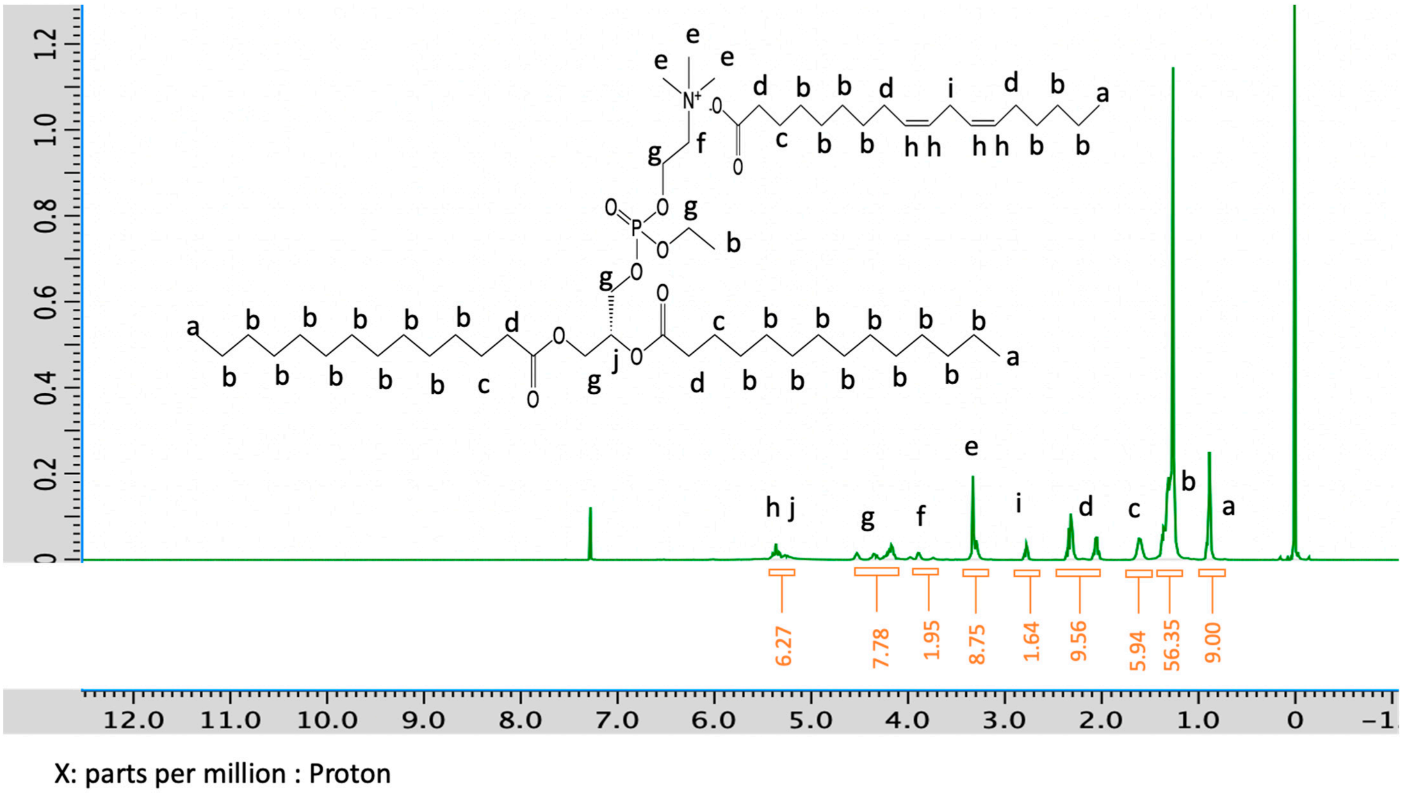

Figure S5. ¹H-NMR of ionic liquid (IL). Spectra was obtained by JEOL ECZ400S 400MHZ NMR, Tokyo, Japan.

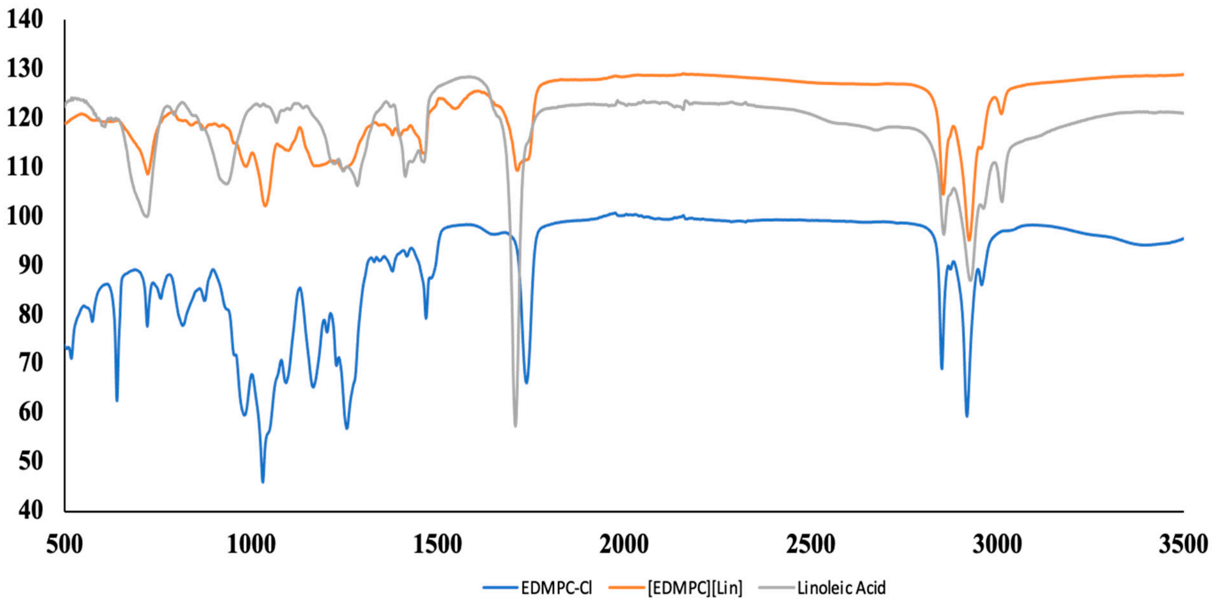

Figure S6. FTIR spectra of 1,2-dimyristoyl-sn-glycero-3 ethyl-phosphocholine (EDMPC), linoleic acid (Lin) and [EDMPC][Lin] IL.

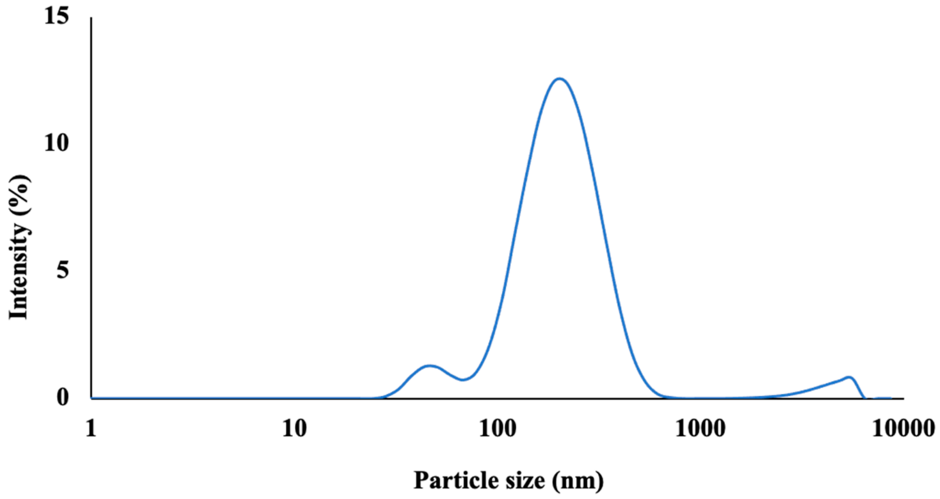

Figure S7. Particle size distribution of IL-S/O. The measured particle size is 174 ± 4.945 nm with polydispersity index of 0.295 ± 0.049
